# Isothermal Amplification Using Temperature-Controlled Frequency Mixing Magnetic Detection-Based Portable Field-Testing Platform

**DOI:** 10.3390/s24144478

**Published:** 2024-07-11

**Authors:** Max P. Jessing, Abdalhalim Abuawad, Timur Bikulov, Jan R. Abresch, Andreas Offenhäusser, Hans-Joachim Krause

**Affiliations:** 1 Institute of Biological Information Processing: Bioelectronics (IBI-3), Forschungszentrum Jülich, 52428 Jülich, Germany; m.jessing@fz-juelich.de (M.P.J.); a.abuawad@fz-juelich.de (A.A.); t.bikulov@fz-juelich.de (T.B.); jan-raphael@abresch.koeln (J.R.A.); a.offenhaeusser@fz-juelich.de (A.O.); 2 Faculty of Mathematics, Computer Science and Natural Sciences, Rheinisch-Westfälische Technische Hochschule Aachen University, 52062 Aachen, Germany

**Keywords:** recombinase polymerase amplification, frequency mixing magnetic detection, thermal lumped parameter model, magnetic nanoparticles, point of care testing

## Abstract

Sensitive magnetic nucleic acid (NA) detection via frequency mixing magnetic detection (FMMD) requires amplified NA samples for which a reliable temperature control is necessary. The feasibility of recombinase polymerase amplification (RPA) was studied within a newly integrated temperature-controlled sensor unit of a mobile FMMD based setup. It has been demonstrated that the inherently generated heat of the low frequency (LF) excitation signal of FMMD can be utilized and controlled by means of pulse width modulation (PWM). To test control performance in a point of care (PoC) setting with changing ambient conditions, a steady state and dynamic response model for the thermal behavior at the sample position of the sensor were developed. We confirmed that in the sensor unit of the FMMD device, RPA performs similar as in a temperature-controlled water bath. For narrow steady state temperature regions, a linear extrapolation suffices for estimation of the sample position temperature, based on the temperature feedback sensor for PWM control. For any other ambient conditions, we identified and validated a lumped parameter model (LPM) performing with high estimation accuracy. We expect that the method can be used for NA amplification and magnetic detection using FMMD in resource-limited settings.

## 1. Introduction

Detection of pathogen contaminations at point of care (PoC), where the traditional central labs are unavailable, is critical for controlling outbreaks and reducing the spread of infectious diseases [1,2]. Among many developed PoC tests, nucleic acid amplification tests have proven to allow precise identification of pathogens with high sensitivity and specificity compared with other techniques [3,4]. However, integrating amplification methods and detecting amplified material in the field remains challenging, particularly when developing a robust, portable and user-friendly assay.

Frequency mixing magnetic detection (FMMD), paired with a portable magnetic reader, offers an accurate and sensitive platform for various applications, particularly in PoC testing [5]. This technology utilizes superparamagnetic nanoparticles functionalized with specific ligands to bind target molecules, generating selective magnetic signals. This allows the detection of various analytes, including antibiotics, toxins, antibodies and pathogens [6]. Compared to other detection methods, the detection of analytes using magnetic labels offers the advantages of selective detection, independent of the sample matrix, which comes from the specific characteristics of superparamagnetic nanoparticles [7]. Moreover, FMMD provides quantitative results for a wide concentration range [8], allowing for precise measurement of analyte concentrations. In addition, it showed the feasibility of multiplex detection of different magnetic nanoparticles [9,10]. Recently, we presented a new magnetic particle assay, that was developed for the detection and quantification of amplified *Brucella* DNA, highlighting the practical use of FMMD in nucleic acid detection [8]. The assay showed a high sensitivity and specificity in detecting amplified DNA with less than 10 min of detection time. However, the detected DNA was amplified by PCR which required a laboratory-based thermal cycler that is not suitable for field testing.

To perform any kind of currently known nucleic acid (NA) amplification technique, temperature regulation of the process is required for efficient amplification. The temperature has a critical impact on overall reaction performance, as the enzyme activity, primer annealing, specificity and sensitivity can be influenced by temperature deviations [11,12]. The temperature regulation is usually achieved by utilizing dedicated thermoregulators such as commercial thermal cyclers. The detection of amplified products is mainly performed using traditional techniques such as gel-electrophoresis for qualitative and semi-quantitative analysis of band intensities or fluorescence-based detection methods like Real-Time (quantitative) PCR (qPCR) and digital PCR (dPCR) for quantitative analysis [13,14,15]. However, in any of these methods, expensive laboratory devices are necessary, approaches are time-consuming and highly trained personnel is required. In addition, these methods are not suitable for resource-limited settings.

Isothermal amplification techniques have revolutionized molecular diagnostics by enabling the detection of specific DNA or RNA sequences with high sensitivity and specificity in PoC scenarios [16,17]. Among these techniques, major attention has been paid to Recombinase Polymerase Amplification (RPA) or Loop-Mediated Isothermal Amplification (LAMP) and many more alternatives to Polymerase Chain Reaction (PCR) [18]. Isothermal techniques offer the advantages of simplicity and speed [4,19]. In addition, they have a constant low operating temperature to support the various enzymatic processes involved in DNA or RNA amplification, which makes integration into a field-detection platform feasible, without the need for expensive sophisticated thermal cycling equipment. The isothermal amplification is usually carried out using simple heating devices such as heat block, water bath, chemical heating and battery powered instruments [20,21,22]. However, additional readout and biosensor methods are required to quantify the amplified DNA, such as fluorometric, colorimetric and electrochemical devices [23,24].

To improve the FMMD technique in terms of mobile nucleic acid testing, the integration of isothermal amplification methods can be a crucial advancement as it increases the mobile capabilities and functionalities of the portable magnetic reader without the need for any sample-preprocessing infrastructure. The magnetic reader sensor unit, henceforth referred to as Measurement Head (MH), consists of a nested configuration of coils that generates low- and high-frequency excitation signals, LF and HF excitation, respectively, and picks up the sample’s magnetic response signal. The thermal energy in the MH is mainly generated by the resistive heat of the low frequency excitation coil that is inherently necessary for FMMD signal acquisition. We hypothesize that this thermal energy can be utilized to drive various biological assays or isothermal amplification sample-pretreatment in the field, if controlled properly. Combining isothermal amplification with FMMD allows the selective magnetic detection and quantification of DNA in a single device without the need of additional instrumentation for the amplification process.

To survey the practicability of this endeavor, we use a simple PWM control approach, utilizing temperature feedback from the LF-coil surface and perform RPA at the sample position of the MH by means of duty cycle control, and assess the basic performance from the controller perspective and from the perspective of its utilization for RPA.

Achieving reliable RPA in a PoC setting is particularly challenging. While for steady state temperatures, a linear regression model might suffice to predict the temperature of the sample, the necessity to assess dynamic temperature behavior and larger temperature ranges needs an approach that is capable of transient temperature prediction for arbitrary ambient conditions. Therefore, we suggest lumped parameter models (LPMs) that describe the temperature behavior at relevant points in the MH for arbitrary ambient condition inputs. Besides the analysis of any thermal influences on the biological pre-processing capabilities of the magnetic reader, this model may also play a role in technological reliability advancements, minimizing temperature-dependent resistance fluctuation and potentially leading to more accurate FMMD signal acquisition.

## 2. Materials and Methods

### 2.1. Reagents

TwistAmp^®^ Liquid Basic kit (Product code: TALQBAS01) was obtained from TwistDx™ Ltd. (Maidenhead, UK). GeneRuler Ultra Low Range DNA Ladder (Product code: SM1213) and DNA Gel Loading Dye (6×) (Product code: R061) were purchased from Thermo Fisher Scientific™ (Langerwehe, Germany). ROTI^®^ Prep PCR Purification (Product code: 8503.2) and Agarose standard (Product code: 3810.3) were obtained from Carl Roth GmbH (Karlsruhe, Germany). RedSafe DNA stain (20,000×) (Product code: 21141) was obtained from Hiss Diagnostics GmbH (Freiburg im Breisgau, Germany).

### 2.2. Frequency Mixing Magnetic Detection

Frequency Mixing Magnetic Detection is a technique used to measure the magnetic response of superparamagnetic nanoparticles or of magnetic beads which exhibit nonlinear magnetic properties by measuring the mixed harmonic distortions in the detected signal [5]. In FMMD, these “mixing” harmonics consist of the HF and the LF component of the two distinct excitation fields that are applied to the sample. Besides the coils used for excitation field generation, a detection coil and an oppositely wound reference coil are used for signal collection. Depending on the magnetic particles’ magnetic moment response to the incident excitation fields, the mixing harmonics change.

The applied LF peak excitation field was 16.4 mT at 63 Hz and the HF excitation field applied was 1.34 mT at 40,545 Hz [25]. These field amplitudes and frequencies were optimized for Synomag^®^-D (70 nm), product code 104-19-701 from Micromod GmbH, Rostock, Germany. The peak current through the LF-coil was 240 mA, yielding a power dissipation of approximately 2.4 W, whereas the HF-coil current of 20 mA led to just 7 mW of heating power, 350-fold lower than the LF power. Thus, the LF excitation chain generates enough heat to be used as a temperature control input, and the HF power is negligible.

### 2.3. Pulse Width Modulation Controller

A 2-point PWM feedback controller for LF-amplitude duty cycle adjustment and therefore controlled heat supply was implemented in the Arduino microcontroller software (version v2.20.2) of the magnetic reader. The feedback temperature sensor used for control was a DS18B20 sensor from Maxim Integrated Products, Inc. (San Jose, CA, USA), mounted on the LF-coil surface inside the measurement head. The suggested control algorithm incorporates two distinct, freely selectable error ranges. A first, wider error range for tuning the temperature in the desired interval and a second, narrower error range within which the temperature is kept during the FMMD measurement process. This makes it generally possible to heat up the amplification environment with maximum power fed to the LF-coil, but then regulate the heat with, e.g., only half maximum power.

**Figure 1:**
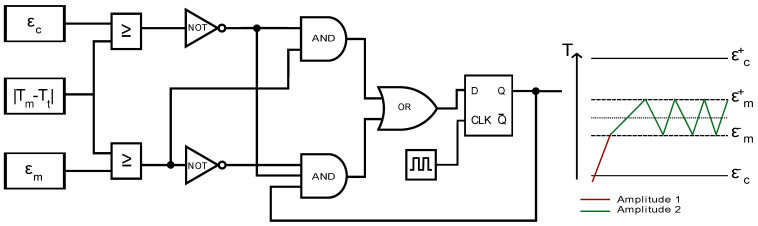
Equivalent logic for a PWM controller with differentially adjustable heating and measurement LF amplitude and schematic of controlled temperature using this logic.

The conditional equivalent logic for the duty cycle regulation within these error ranges based on the temperature difference between the measured output temperature and the set desired temperature input value followed the scheme in Figure 1 below:

Here, ε_c_ denotes the allowed temperature error range during heat up and tuning and ε_m_ denotes the allowed temperature error range during the FMMD measurement. T_m_ is the measured temperature at the LF-coil surface and T_t_ is the set controller target temperature. The output of this equivalent logic states the condition that determines if the LF-amplitude is turned on or off with a first amplitude setting (Amplitude 1) or a second amplitude setting (Amplitude 2), different from the first one. Software-wise, this logic was implemented into the Arduino microcontroller using multi-tasking programming with a time-slicing scheduling technique to account for the wait time of the temperature sensor and measurement readout. 

### 2.4. Recombinase Polymerase Amplification (RPA)

The RPA for positive control reactions was performed following the manufacturer’s recommendation in the TwistAmp^®^ Liquid Basic kit, with slight adjustments by reducing the reagent quantities to create a total volume of 25 µL. In the RPA reaction, a master mix was prepared containing 3.5 µL of oligo mix primers, 12.5 µL of 2× reaction buffer, 2.5 µL of 10× basic E-mix, 2.75 µL of dNTPs and 1.25 µL of 20× core reaction. After mixing, 2 µL of MgOAc and 1 µL of positive control DNA were added to start the reaction. The RPA reaction was carried out at desired incubation temperatures and time inside the measurement head.

After the amplification, the RPA was purified using ROTI^®^ Prep PCR Purification kit through column centrifugation to remove reaction components. For the visualization of RPA products, 10 µL of the purified amplicons was analyzed using agarose gel electrophoresis on 2% agarose gel in 1× TBE buffer at 100 V for 1 h. The visualization was conducted with the ChemiDoc™ XRS Imaging System (Bio-Rad Laboratories Co., Ltd., Hercules, CA, USA).

### 2.5. Lumped Parameter Model

#### 2.5.1. Model Structure Design

The model structure was designed using a grey box approach. The following assumptions (logical derivations based on physical intuition or measurement) were made:

1. The LF power contributes significantly more to MH heating than the HF-coil power. This assumption is based on the 350-fold difference in power dissipation described in Section 2.2.
2. The ambient temperature influences the heating and cooling processes of the MH (heat dissipation is influenced by the temperature difference between the system under test and its surroundings—Newton’s law of cooling, Stefan–Boltzmann law).
3. From 1 and 2, it follows that the model has two inputs (ambient temperature and average LF power).
4. Heat transfer is a non-integer order process (hence, Padé approximations in the heating and cooling lanes).
5. The MH can store a certain amount of heat energy (it has a heat capacity, respectively) denoted by $C_{vol}$.
6. At the sample position, the system obeys a transport delay term that describes the time the heat needs to travel from the sensor to the sample position.

The simulation was set up using MATLAB Simulink (version R2023b). As a numerical method for solving the model equations, the auto-select Bogacki–Shampine solver was used. For the “feedback position model”, a fixed step size (fundamental sample time) of 0.061 s for the system identification dataset and 1.688 s for the validation dataset was used. Here, system identification is the model parameter estimation process for measured in- and output data compared to the model output, such that the deviations between measured system output and model output are minimized. System validation is a repetition of this process for independently recorded different in- and output data and the confirmation that the model captures the essential system dynamics. The total length of the validation dataset was 21,111 s and the length of the identification dataset was 3042 s. Considering the trade-off between simulation accuracy and computational effort for parameter estimation, the difference in auto-selected fixed-step size was accepted. The model validity was not influenced. The “sample position model” was solved using variable step sizes to significantly reduce estimation time for initial delay time estimations. The identification dataset for this model was recorded over 5720 s and the validation dataset was recorded over 10,997 s. For each model identification and validation recording, different lengths of averaged power input pulses were used.

#### 2.5.2. Model Parameter Estimation (Model Parametrization)

The free model parameters ($K_{LF},K_{AMB},C_{vol}$, $a_{1}–d_{5}$) were estimated by solving a least squares optimization problem. Its cost-function definition can be seen in Equation (1), where y_p_ is the predicted value and y_m_ is the measured value.

(1) $$J=\min\sum_{i=1}^{n}{\left(y_{p}-y_{m}\right)}^{2}$$

This optimization problem was solved using the parameter estimator of the system identification toolbox in MATLAB, which iteratively combines different parameters and compares a measured dataset to the model output for each iterative set of parameters. This process is repeated until a cost-function minimum is reached.

### 2.6. Model Performance Metrics

To compare the measured data to the simulated model output, the Normalized Root Mean Square Error (NRMSE) was determined. Normalization can be performed with respect to the mean of the measured response data or with respect to the range of data points (y_max_–y_min_). Depending on whether the NRMSE is normalized with respect to the mean or the range of the measured dataset, NRMSE is sometimes also called the coefficient of variation (CV).

The latter was used in this study and the NRMSE was calculated according to Equation (2).

(2) $$\mathrm{NRMSE}=\frac{\sqrt{\frac{1}{n}\sum_{i=1}^{n}{\left(y_{m_{i}}-y_{p_{i}}\right)}^{2}}}{y_{m_{\max}}-y_{m_{\min}}}$$

with the number of measurements, *n*, the measured value $y_{m_{i}}$ and the predicted value $y_{p_{i}}$.

The residual variance between the measured data and the simulated output for an estimated set of model parameters is lower when the NRMSE value is small.

Furthermore, considering a predictor variable (number of inputs to the system) adjusted statistical metric, the standard Error of Estimate (SEE) was used in the linear/multi-linear models. Closely related to the RMSE, it accounts for the degrees of freedom in the system,

(3) $$\mathrm{SEE}=\sqrt{\frac{\sum_{i=1}^{n}({y_{m_{i}}-y_{p_{i}})}^{2}}{n-p}}$$

with p being the predictor variable, accounting for the number of independent variables in the system under test. Accordingly, for additional intuitive depiction, the % agreement was calculated based on the adjusted R^2^ value.

## 3. Results and Discussion

### 3.1. Controller Performance

**Figure 2:**
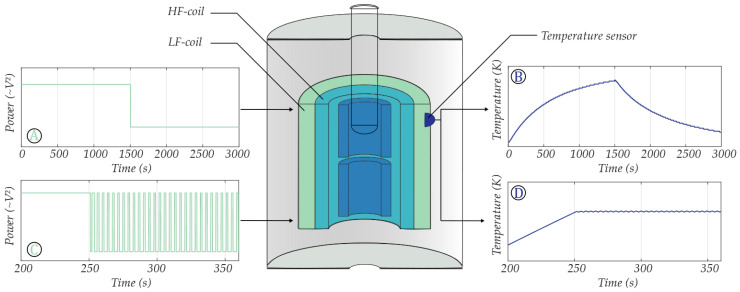
Schematic of the measurement head cross section, including the temperature sensor position. (**A**) Uncontrolled low frequency power input step. (**B**) Uncontrolled temperature output corresponding to the input depicted in (**A**). (**C**) Pulse width modulated low frequency power input. (**D**) Adjustable temperature output corresponding to the PWM input depicted in (**C**).

Before testing the feasibility of RPA in the MH, the temperature controller should briefly be investigated in terms of its reliability in a test (lab) setting. While performing any NA amplification as well as during FMMD signal acquisition, the sample to be amplified or measured is inside the sample bore of the MH. Due to geometrical restrictions, sample and temperature sensors cannot be installed simultaneously at the current stage. Hence, the temperature sensor used for feedback control was mounted on the surface of the LF-coil in the MH, a small distance apart from the center of the sample position. To visualize this, a schematic of the MH, the temperature sensor and sample position, as well as the principle of the power to temperature conversion can be seen in Figure 2.

**Figure 3:**
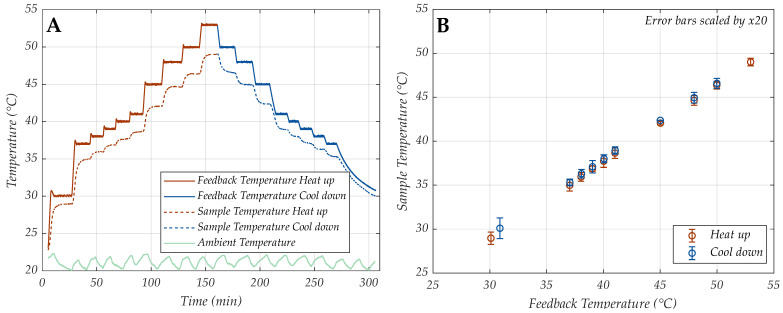
Recorded characterization data for feedback temperature control, sample position temperature and ambient (laboratory) temperature of 21 °C (**A**) and linear dependency of controller temperature vs. sample position temperature (**B**). The standard deviation bars in B are scaled by 20× and indicate the stability of the temperature control at sample position.

Because of distinct control and amplification locations, it is important to not only characterize the controller and controllable temperature ranges at the position of the feedback sensor, but especially to test the temperature control at the sample position that is not directly controlled. The feedback sensor behavior was characterized for 10 different feedback temperature settings (30, 37, 38, 39, 40, 41, 45, 48, 50, 53 °C) set in ascending (heating) and descending (cooling) order between room temperature and 53 °C (Figure 3A, solid lines). The corresponding temperatures at sample position were recorded with an epoxy-passivated temperature sensor in DI-water simultaneously (Figure 3A, dashed lines).

The heating and cooling profiles at the sample position were determined for several different steady ambient conditions at 16, 18, 21 and 23 °C, similarly. The graphs for 16, 18 and 23 °C ambient temperature can be found in the Supplementary Materials Figure S1, they were additionally used for control performance estimations.

To determine the sample position temperature for any feedback controller setting, first a “calibration” was performed. The relation of the temperature at the region of interest (sample position) was plotted against the temperature at the more accessible LF surface location, suited for controlling (Figure 2B). This way, a linear relationship of $T_{s}$ (sample temp.) and $T_{f}$ (feedback temp.) could be ensured to easily select the necessary amplification temperatures at the sample position.

To initially estimate the reliability, the measured data at the recorded ambient temperatures were used to test the PWM controller strategy (at the sample position, where the amplification happens) in terms of heat up/cool down time constant, hysteresis and temperature control stability.

The heat up and cool down time constants $\tau_{h_{16–23}}$ and $\tau_{c_{16–23}}$ were determined by exponential fits for each step of the heating and cooling data, respectively (plots can be found in Supplementary Figures S2 and S3). The temperature data were fitted from their inflection point to the last point of their saturation by:

(4) $$T_{h}\left(t\right)=K_{h}\cdot (1-e^{\frac{-\left(t-t_{d}\right)}{\tau_{h}}})$$

The cooling data were fitted similarly with a decreasing exponential:

(5) $$T_{c}\left(t\right)=K_{0}+K_{c}\cdot e^{\frac{-\left(t-t_{d}\right)}{\tau_{c}}}$$

where for the heating process fit, $T_{h}\left(t\right)$ eventually approaches the asymptotic value $K_{h}$. For the cooling process fit, $T_{c}\left(t\right)$ eventually approaches the offset-term $K_{0}$, while $K_{c}$ indicates the initial deviation from $K_{0}$. The time $t_{d}$ is the initial time and $\tau_{h/c}$ represents the time constant.

The average heating and cooling time constants were calculated to be $\tau_{h_{16–23}}=137.13\;s$ and $\tau_{c_{16–23}}=158.59\;s$, respectively. Therefore, for all characterized 1–7 °C step cases, 2/3 of the steady state signal was reached in less than 2.3 min for heating and 2.7 min for cooling.

The hysteresis (here, the difference in temperature level for each step of the heating process compared to the corresponding step of the cooling process) of both, the feedback control temperature and the sample position temperature were determined for the same datasets. The average hysteresis of the PWM controller at feedback temperature position was calculated to be below the resolution limit of the digital DS18B20 temperature sensor (<0.0625 °C) and therefore was not considered. The hysteresis of the temperature control at the sample position averaged 0.29 °C (>4× resolution of DS18B20). Although temperature control accuracy is crucial to ensure consistent amplification yield, RPA is generally feasible within a relatively large temperature span (37–42 °C). We defined a requirement of a 1–2 °C fluctuation at maximum, while in any case staying below 42 °C, which could denature RPA components. The determined hysteresis at the sample position is therefore negligible.

**Figure 4:**
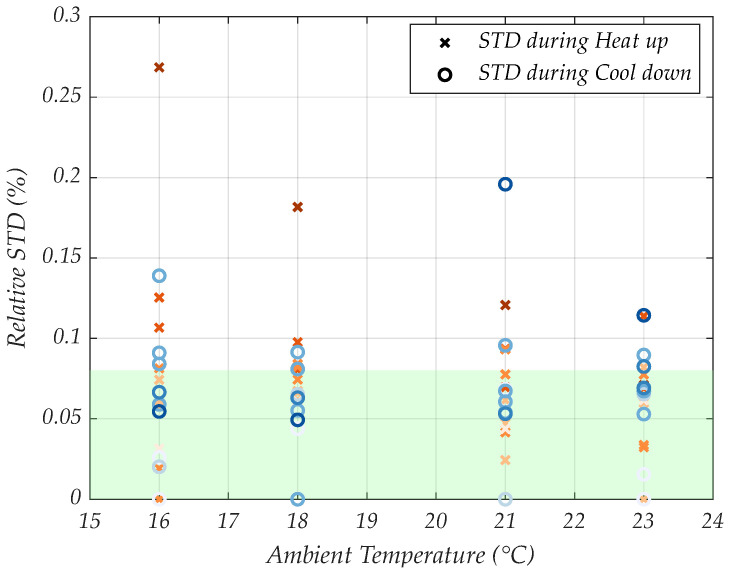
Relative standard deviations for steady state data at four different ambient temperatures.

After reaching the desired steady temperature at the sample position, the importance of control stability becomes obvious. Therefore, the temperature stability of each temperature step was determined by means of its relative deviation from the mean over a 10 min time period (Figure 4), equivalent to the time that is at least needed for a recombinase polymerase amplification process [26,27].

The orange crosses indicate the % change from the set temperature control during all heat up steps from Figure 3A, while the blue circles indicate the same during the steady state phases of the cool down steps. The more intense colors mark higher temperature levels. The relative standard deviation (Figure 4, y-axis) was investigated at the previously described ambient conditions (Figure 4, x-axis). The green-colored region indicates the range of errors within the region of temperatures that are most appropriate in an RPA process, which is 37–42 °C, respectively.

From this plot, we obtain three important facts:

- As the temperature difference between the set controller value and the ambient increases, the stability error tends to increase.
- Higher controlled temperatures generally yield larger stability errors.
- For all investigated constant ambient conditions, a stability error of maximally 0.3% was observed.

Since we want to prove feasibility first, and optimize later, let us just consider the third point for now. A marginal error of 0.3% would amount to 0.11 °C. Considering that the error in the later selected RPA operating temperature region from 37–42 °C stays below a 0.1% error, the temperature instability during the amplification process will not influence the efficiency of RPAs significantly.

### 3.2. RPA Amplification

To test the functionality in terms of amplifying DNA at the sample position, we performed RPA in our temperature-controlled measurement head. The selection of RPA was based on its rapid amplification time compared with others techniques like LAMP, RCA, NASBA with similar sensitivity and specificity [18]. The typical incubation time of RPA is between 20 and 30 min [28]. However, several studies showed the capability of RPA to amplify DNA in less than 10 min [26,27]. We achieved rapid and efficient amplification using our portable magnetic reader. By combining RPA with our previous work, which demonstrated the ability to rapidly detect amplified DNA in less than 10 min [8], a PoC analysis system can be suggested that does not require any sample pre-treatment in laboratories.

As a proof of concept, we performed RPA amplification using the positive control template and oligo mix primers provided in the TwistAmp^®^ Liquid Basic kit, which is expected to produce an amplicon of 289 bp. The RPA reaction operates at constant temperature, typically between 37 °C and 42 °C. This temperature range is based on the optimal activity of the enzymes involved in the reaction such as recombinase enzymes, polymerases and other components. In our study, we tested the amplification across a range of temperatures, including 21 °C, 30 °C, 37 °C, 38 °C, 45 °C and 50 °C. The selection of temperatures was chosen to investigate the amplification efficiency both within and outside the optimal operating temperature range of RPA. This way, we could assess how the variations of temperature controlled by PWM affect the performance of RPA amplification from the biological perspective. The amplification was done both inside our temperature-controlled measurement head and in a water bath as a reference method. In both cases, the temperature ranged from 30 °C to 50 °C with an incubation time of 30 min.

**Figure 5:**
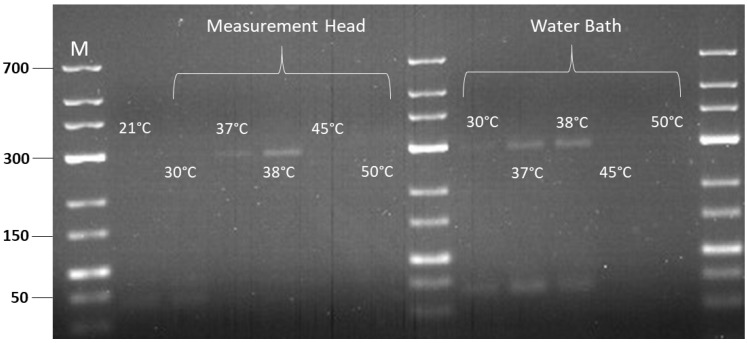
Gel image of RPA amplification inside measurement head and water bath at different incubation temperatures. M: Marker.

Figure 5 shows the gel image of the RPA products amplified inside our measurement head and in a water bath, controlled to different temperatures. From the gel image, we confirmed the successful RPA positive control amplification as the expected amplicons with a size of 289 bp were observed at 37 °C and 38 °C in both our measurement head and water bath. When the amplification was tested outside the operating temperature of RPA, such as at low temperatures (21 °C and 30 °C) and at high temperatures (45 °C and 50 °C), no bands were observed. This can be explained by the decreased activity of the enzymes at lower temperatures and enzyme denaturation at higher temperatures, which result in inefficient amplification and the absence of bands on the agarose gel. From this result, we conclude that our implementation of a PWM controller regulated and controlled the temperature of the measurement head sufficiently, as the bands were observed at the optimal temperature range of RPA (37 °C and 38 °C), while no bands were observed at low and high temperatures.

**Figure 6:**
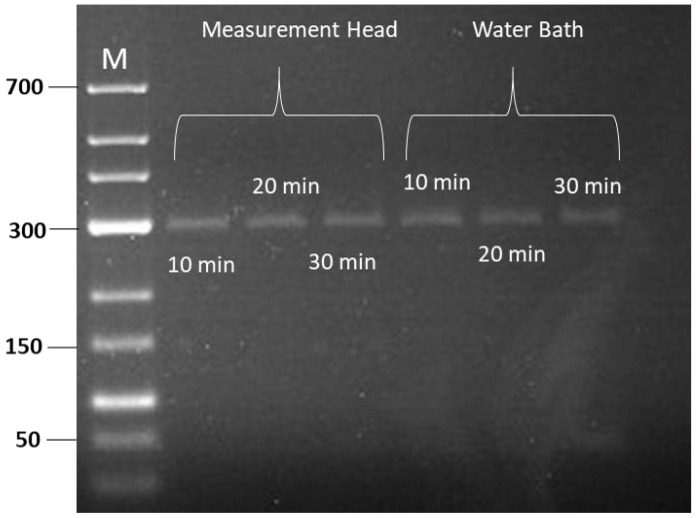
Gel image of RPA amplification inside measurement head and water bath at different incubation times. M: Marker.

To validate the temperature stability at the sample position in terms of the amplification performance, we performed RPA at different incubation times ranging from 10 to 30 min. Similar to previous investigations, the amplification was executed inside our measurement head and in a water bath at 38 °C. After the amplification, the amplified products were purified and loaded into the gel for 1 h. As shown in Figure 6, the band intensities for the RPA products amplified inside the measurement head and water bath were similar at all incubation times. This confirms that the stability of the temperature controlled by PWM inside the measurement head and the water bath control performance are alike in terms of their potential for successful RPA.

### 3.3. Linear Extrapolation Model

After evaluating the PWM control approach and proving the feasibility of RPA in the MH unit, we want to examine the applicability of RPA in our portable magnetic reader device.

Based on the previously presented results, the requirements for a successful RPA in the measurement head were defined as:

1. The controlled temperature at the sample position cannot exceed 42 °C in any case, as amplification components may denature.
2. The PWM controller reached a temperature stability error of less than 0.3% at the sample position. With the successfully executed RPA presented in this section, this stability is exceedingly sufficient.
3. A maximum control error of ±1 °C was stipulated at the sample position, as the RPA worked well at 37 °C and 38 °C.

**Figure 7:**
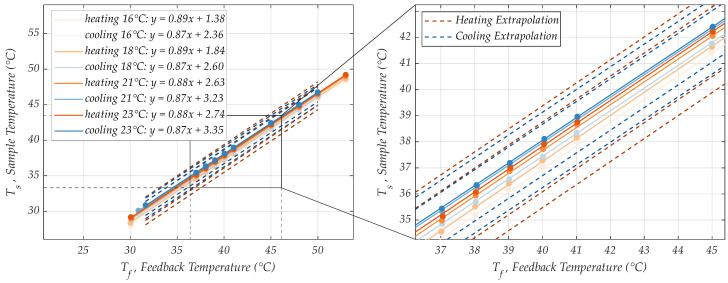
Linear heat-up and cool-down relationship of $T_{s}$ and $T_{f}$ at 16, 18, 21 and 23 °C ambient temperature. And the same linear relation zoomed in to the region of RPA operating temperatures with linear model-estimated calibration curves for 10, 13, 26 and 29 °C ambient temperature.

To first examine the applicability of RPA in the MH in a smaller temperature working range (16–23 °C), a linear extrapolation model may suffice. The starting points for linear extrapolation are the $T_{s}$/$T_{f}$-relation curves, exemplarily shown in Figure 3B. Similarly, these curves were recorded for the heating and cooling process of other previously investigated ambient temperatures (Figure 7).

While the slopes of the linear regression of the $T_{s}$/$T_{f}$-relation curves only change marginally for all investigated ambient conditions (see equations in Figure 7), the $T_{s}$-axis intercept seems to linearly depend on the ambient temperature level. For this model, we assume that this linear dependency persists for arbitrary but steady ambient temperature values. Therefore, linear extrapolation can be performed, estimating $T_{s}$/$T_{f}$-relation curves for different steady ambient temperature levels. Due to the negligibly small slope changes, we averaged the slopes of heating and cooling curves, respectively. This slope can be kept for all predicted $T_{s}$/$T_{f}$-relation curves. The underlying linear relation between the y-axis intercept and ambient temperature can be described for the heating process by $T_{s,intercept,h}=0.2T_{f,h}-1.85$ and for the cooling process by $T_{s,intercept,c}=0.15T_{f,c}-0.08$, which was obtained by mapping the intercept value versus the ambient condition and fitting a linear function.

To test the extrapolation approach, datasets for 16, 18 and 21 °C were considered and the “calibration curve” data for 23 °C ambient temperature was estimated using the model. Subsequently, we compared the data estimated by the model to the measured data points. The real intercept of the measured data was 2.74, while the predicted intercept with the linear extrapolation model was 3.12. For this data sample, the measured data matches the predicted data with an R^2^ value of 0.96 and a standard error of estimate (SEE) of 0.79 °C. With the requirements for a successful RPA in the MH defined earlier, the estimation error in our model should be <0.89 °C, taking into account the maximum control fluctuation of 0.11 °C at the sample position. Hence, these statistical metrics confirm that an accurate enough prediction is indeed possible relative to the measured data in the proposed steady state ambient temperature range.

Therefore, combining the PWM approach and FMMD technology with this linear regression model provides a simple, yet functional way to control the temperature for sample pre-processing. Moreover, the magnetization response of a magnetic nanoparticle-based immunoassay or DNA assay can potentially even be recorded simultaneously to temperature regulation, during the “on-time” of the duty cycle.

While PWM and the linear regression model already meet all necessary requirements for RPA in the investigated environment, the main limitation of the model is its steady state assumption for ambient temperature conditions around room temperature. Estimating error margins for predicted calibration curves turns out statistically insignificant without testing the control approach in many different environmental conditions first. Additionally, conclusions 1 and 2 from Figure 4 indicate that, while stability errors are small for the presented data of the temperature at the sample position, they still depend on the strength of the ambient temperature level-shift.

### 3.4. Thermal Lumped Parameter Model

To overcome the steady state assumption of the extrapolation model, lumped parameter model (LPM) systems were identified and validated for measured temperature data $T_{f,meas}$ at the LF-coil surface and for measured data $T_{s,meas}$ at the sample position. Using the LPM approach, we can evaluate the transient thermal behavior of our system for arbitrary ambient temperature inputs at the points of interest. By connecting both models, control performance can also be estimated at the sample position, despite the fact that the feedback temperature is tracked at the LF-coil surface.

Of course, in reality the MH underlies a spatially varying temperature distribution that changes radially throughout its cylindrical shape. The zero-dimensional assumption of the lumped parameter model considers solely the transient temperature behavior. In other words, this model structure is limited to predicting a single point temperature. However, since the MH geometrically obeys the same physics laws everywhere, with the only difference being material constants or different transport delays, minor adjustments in the model can be used for the prediction of different single point temperatures in the MH, like $T_{f,pred}$ and $T_{s,pred}$.

The LPM interconnects the temperature output of the system $T_{f}$ (LF-coil temperature) or $T_{s}$ (sample position temperature in water), respectively, to the average dissipated power from the LF-coil ($\bar{P_{LF}}$) and the ambient temperature ($T_{AMB}$) through an effective thermal capacitance term $\frac{1}{C_{vol}}$. A feedback loop of the output temperature to the $T_{AMB}$ input shows the cooling/heating dependence on the temperature difference of the MH and its surroundings. To explain this, let us first introduce the system represented as the underlying differential equation:

(6) $$\frac{{dT}_{f}(t)}{dt}+p_{c}^{*}\frac{K_{AMB}}{C_{vol}}T_{f}(t)=p_{h}^{*}\frac{K_{LF}}{C_{vol}}\bar{P_{LF}}(t)+p_{h}^{*}\frac{K_{LF}}{C_{vol}}T_{Amb}(t)$$

The term $C_{vol}\cdot \frac{dT_{f}(t)}{dt}$ may remind one of the energy storage term in the heat conduction equation without the spatial dependencies of thermal diffusivity—density and thermal conductivity. It is the core piece that allows transient analysis. $K_{LF},K_{AMB},C_{vol}$ are the main parameters in the model. $K_{LF},K_{AMB}$ scale the influence of the system inputs $\bar{P_{LF}}$ and $T_{AMB}$ on the MH’s thermal output behavior. Generally, the right side of the equation is a function, describing the inputs of the system, equivalent to the standard heat conduction description.

**Figure 8:**
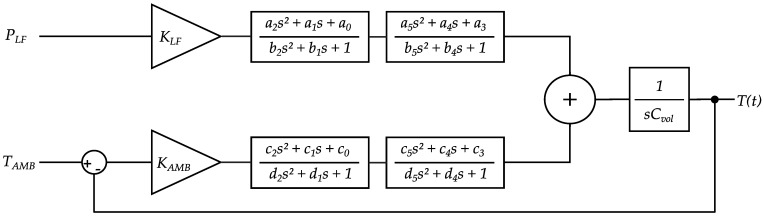
Block diagram of a lumped parameter model for the LF-coil surface temperature.

Based on the heat conduction equation supplemented by the assumptions defined in Section 2.5.1, the model structure was iteratively determined. A more intuitive way to depict this system and a typical approach in control theory is the illustration as a block diagram. Each part in the block diagram represents either an input, an output, a constant or a Laplace-transformed portion of the underlying differential equation that determines the systems dynamics. Figure 8 shows the block diagram that represents the thermal behavior of the MH in time.

In this block diagram, s is called the complex frequency variable (or Laplace parameter) resulting from the Laplace transform, representing time derivatives at the order of the potency of s. The linearity of each transfer function in this first model structure allows the scaling of the input measures for achieving proportional changes in the model output. As can be seen from the differential form of the model, this is fundamentally a linear first order multi-input, single output (MISO) system.

Two modifications will be made to this general model structure before we can identify and compare the $T_{f}$ and $T_{s}$ output confidently.

1. It was reported that heat conduction can be more accurately described by a non-integer order process [29]. So, we attempted integer-order polynomial approximations (Padé approximations) to model the non-integer order portion of the problem and enhance the accuracy of the model output compared to measured data. The higher the order of approximation, the more accurately the model output reflects the measured data. In one of the present cases, for example, a fourth order Padé approximation improved model accuracy in terms of R^2^ value by ~13% over a second order Padé approximation. This improvement diminishes strongly with higher approximation orders. The trade-off involves that a higher number of parameters in the model is needed, and the model requires higher computational parameter estimation effort and therefore more time [30]. Exemplary second order Padé approximations follow the form:

(7) $$p_{i}^{*}=\frac{a_{2}s^{2}+a_{1}s+a_{0}}{b_{2}s^{2}+b_{1}s+1}$$

where $p_{i}^{*}$ denotes the Padé block, with i being representative of the approximation purpose (heating or cooling influence, respectively) and $a_{0}-b_{2}$ are constants. We decided to use two blocks of second order Padé approximations to “smoothen” the effect of each of our inputs $P_{LF}$ and $T_{AMB}$ to the output $T_{s,pred}$ or $T_{f,pred}$. The reason for using two blocks of second order approximations instead of one block of fourth order was simply that the computational effort is substantially reduced for two separate blocks in MATLAB.

2. In order to extend the described model structure to be usable as a sample position model instead of determining solely the LF-coil temperature, a transport delay term was multiplied with the system, using a delay term block in MATLAB Simulink. Typically, transport delays occur as nonlinear elements in a first instance. However, the sample position temperature model is consequentially time delayed to the LF-coil temperature model, and hence is nonlinear; both models remain linear relative to the $T_{AMB}$ and $\bar{P_{LF}}$ inputs. The scaled transport delayed temperature output at the sample position can therefore be considered as an output on the LF-coil surface. Linearization of the delay time, which could classically be done by Padé approximations as well, can be omitted for now, which reduces the number of parameters in the model and makes simulations faster.

In the course of system identification, $K_{LF},K_{AMB},C_{vol}$ and the Padé approximation parameters were determined solving a least squares problem (7) and were collected in Table 1. Using the identified parameters, the models were validated with additional independently recorded datasets at sample and feedback position, respectively. The corresponding measured and simulated system inputs and outputs are depicted in Figures S4–S7 in the Supplementary Materials.

To evaluate the model fit accuracy, selected statistical metrics were introduced in Section 2.5.2 and can be seen in Table 2 for the various identification and verification datasets for the feedback model with the output $T_{f,pred}$ and the sample position model with the output $T_{s,pred}$.

Overall, the evaluation indicates very good model performance, with small error margins. Due to the relatively slow nature of temperature change, the model is able to capture the essential thermal dynamics of the MH sensor unit.

**Figure 9:**
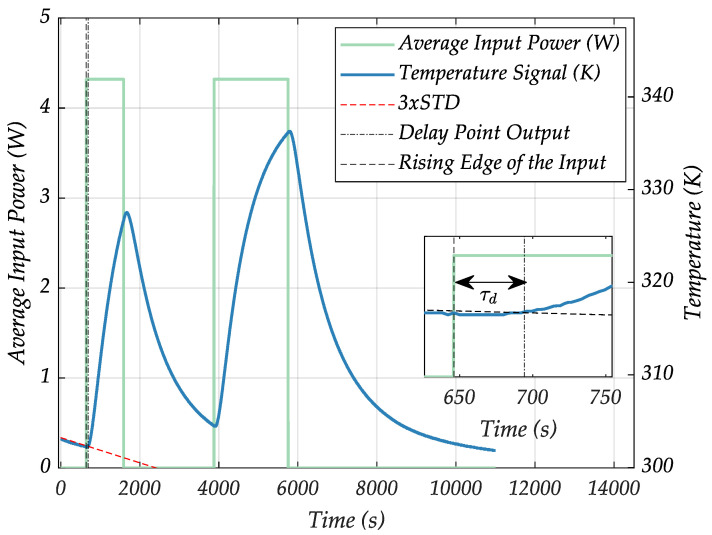
Delay time estimation for the sample position LPM. The red line indicates a slope-adjusted regression line at three times the standard deviation of the initial piece of the measured dataset. The green curve indicates the average input power to the system that causes the temperature output behavior (blue curve).

The long transport delay time between the LF-coil and the sample position was estimated to be $\tau_{d}=48.27\;s$. It was determined at what time measured temperature data first exceeded 3× the standard deviation of a linear regression line fitted to the initial linear data of the identification and validation datasets. To illustrate this more clearly, an example of a delay-time estimation based on the validation dataset of the sample position model can be found in Figure 9.

**Figure 10:**
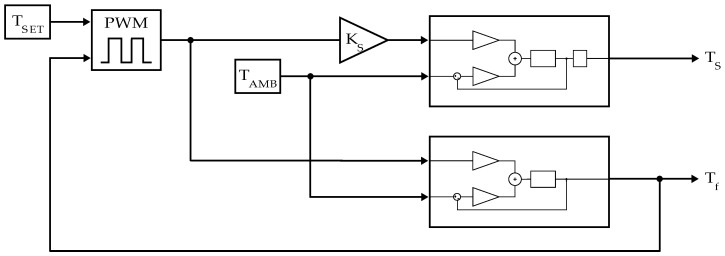
Combined model system block diagram with PWM controlled average power input, T_SET_ the desired control temperature and K_S_ a scaling factor for the control action at the sample position.

Finally, the last step was to connect the model structures of the LPM for $T_{f,pred}$ and $T_{s,pred}$ by using the controlled $P_{LF}$ output of any controller (here, PWM is an example), generated based on the temperature feedback from the LF-coil surface and feed it to both LPMs as the controllable input (see Figure 10). The $T_{Amb}$ input is also similar in this case for both models. 

**Figure 11:**
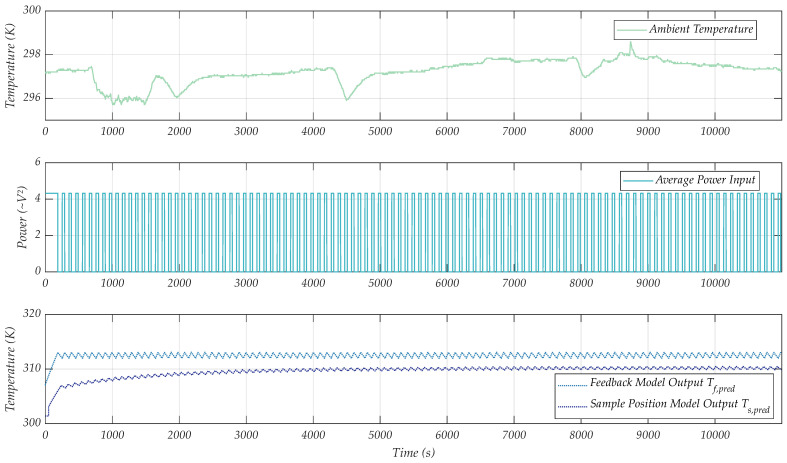
Ambient temperature input profile (**top**), duty cycle of the average power input (**mid**) and controlled temperature at the feedback position (blue) and sample position (purple) (**bottom**).

Doing this, the connected model presents the simulated control output for both the feedback position temperature $T_{f,pred}$ and the sample position temperature $T_{s,pred}$ (Figure 11), exemplarily for 38 °C, a typical RPA operating temperature. The initial condition fed to the integrator of the models need to be distinct in this case—the LPM for feedback position uses the initial value of the feedback temperature and the LPM for sample position uses the temperature at the sample position at the start of the measurement. As was observed in the measured data, the temperature control at the sample position is a delayed and scaled version of the temperature at the LF-coil surface. By introducing a simple scaling factor $K_{S}$ to the input of the sample position model, we are able to predict these temperature curves at different MH positions for arbitrary ambient inputs. We propose that this enables the selection of sensible control approaches, depending on the severeness of ambient conditions in the field, and to further identify physical limitations for biological use cases.

## 4. Conclusions

The feasibility of performing recombinase polymerase amplification in the measurement head of the magnetic reader device prior to FMMD signal acquisition at continuously constant ambient conditions was presented using a PWM approach with the inherently generated heat from the LF excitation coil of the system. This simple implementation has already proven to be a valuable addition to the mobile functionality of the FMMD device for the RPA-based sample preparation.

A linear relation of the sample temperature to the temperature at the position of the feedback sensor determined the necessary control temperature to set. Despite the sample opening in the MH, a level-shift in the ambient conditions leads to a shift of the *T_s_*/*T_f_*-relation curve towards the new ambient temperature state (Figure 7). While the feedback temperature *T_f_* stayed constant (as it was embedded within the MH and isolated from the environment), the sample position temperature shifted slightly for temperatures between 16 and 23 °C. To investigate the reliability of the PWM approach at ambient conditions different from the tested ones, a linear regression model for prediction of the steady state and an LPM for prediction of the transient behavior of the temperature at the sample position for arbitrary ambient condition inputs was suggested. The latter does not only help in investigating thermal behavior of the MH at the sample position for arbitrary inputs, it is also a promising tool for future technological advancements. It may help in minimizing resistance fluctuations that have an effect on the excitation signal amplitudes or in determining the thermal capacitance of the entire MH for 3D analysis.

## Figures and Tables

**Table 1:** Estimated model parameters for LPM at feedback and sample position.

Model Parameters	LPM LF-Coil	LPM Sample Pos.
$K_{LF}$	0.302	0.317
$K_{AMB}$	0.052	0.046
$C_{vol}$	36.266	45.572
$a_{0}-a_{5}$	[1.103, 1.279, 0.001, 1.145, 1.139, 1.000]	[1.142, 1.305, 0.001, 1.048, 1.165, 1.000]
$b_{1},b_{2},b_{4},b_{5}$	[0.737, 1.011, 0.877, 0.980]	[0.730, 1.011, 0.870, 0.980]
$c_{0}-c_{5}$	[0.892, 0.999, 0.999, 0.839, 0.996, 1.018]	[0.806, 0.998, 0.999, 0.864, 0.995, 1.018]
$d_{1},d_{2},d_{4},d_{5}$	[1.007, 1.007, 0.987, 0.997]	[1.010, 1.007, 0.988, 0.997]

**Table 2:** Statistical measures to evaluate the simulation quality based on measured data.

Metrics	$T_{f,pred}$ Identification/ Validation	$T_{s,pred}$ Identification/ Validation
NRMSE	0.049/0.075	0.047/0.066
$\%—\mathrm{Fit}\;\mathrm{based}\;\mathrm{on}\;R_{adj}^{2}$	96.91%/96.04%	95.28%/93.45%
SEE	1.523/1.600	1.693/2.257

## Data Availability

The original contributions presented in the study are included in the article and in the Supplementary Materials, further inquiries can be directed to the corresponding author.

## Supplementary Materials

The following supporting information can be downloaded at: https://www.mdpi.com/article/10.3390/s24144478/s1, Figure S1: Recorded characterization data for feedback temperature control, sample position temperature and ambient (laboratory) temperature of 16, 18 and 23 °C; Figure S2: Exponential fit and time constant estimation of the standard deviation cured heating steps in the temperature characterization data (21 °C) from Figure 3; Figure S3: Exponential fit and time constant estimation of the standard deviation cured cooling steps in the temperature characterization data (21 °C) from Figure 3; Figure S4: System in- and outputs used as model structure identification data for the lumped parameter model that predicts temperatures at the feedback sensor position; Figure S5: System in -and outputs used as model structure validation data for the lumped parameter model that predicts temperatures at the feedback sensor position; Figure S6: System in- and outputs used as model structure identification data for the lumped parameter model that predicts temperatures at the sample position; Figure S7: System in- and output used as model structure validation data for the lumped parameter model that predicts temperatures at the sample position; Figure S8: Delay time estimation for system identification dataset. The delay time is the difference of the rising edge of the input pulse and the first significant change in the output temperature.
