# Supplementary material for: Isothermal Amplification Using Temperature-Controlled Frequency Mixing Magnetic Detection-Based Portable Field-Testing Platform
Source: Sensors (Basel). 2024 Jul 11;24(14):4478. doi: 10.3390/s24144478 (PMC11281083; doi:10.3390/s24144478)
Supplement: Supplementary file 1 [file sensors-24-04478-s001.zip › sensors-3072427-supplementary.pdf]

Supplementary Material

Isothermal Amplification Using Temperature-Controlled Frequency Mixing Magnetic Detection-Based Portable Field-Testing Platform

Max P. Jessing [1,2,†], Abdalhalim Abuawad [1,2,†], Timur Bikulov [1,2], Jan R. Abresch [1], Andreas Offenhäusser [1,2] and Hans-Joachim Krause [1,*]

1 Institute of Biological Information Processing: Bioelectronics (IBI-3), Forschungszentrum Jülich, 52428 Jülich, Germany; m.jessing@fz-juelich.de (M.P.J.); a.abuawad@fz-juelich.de (A.A.); t.bikulov@fz-juelich.de (T.B.); jan-raphael@abresch.koeln (J.R.A.); a.offenhaeusser@fz-juelich.de (A.O.)

2 Faculty of Mathematics, Computer Science and Natural Sciences, Rheinisch-Westfälische Technische Hochschule Aachen University, 52062 Aachen, Germany

* Correspondence: h.-j.krause@fz-juelich.de

† These authors contributed equally to this work.

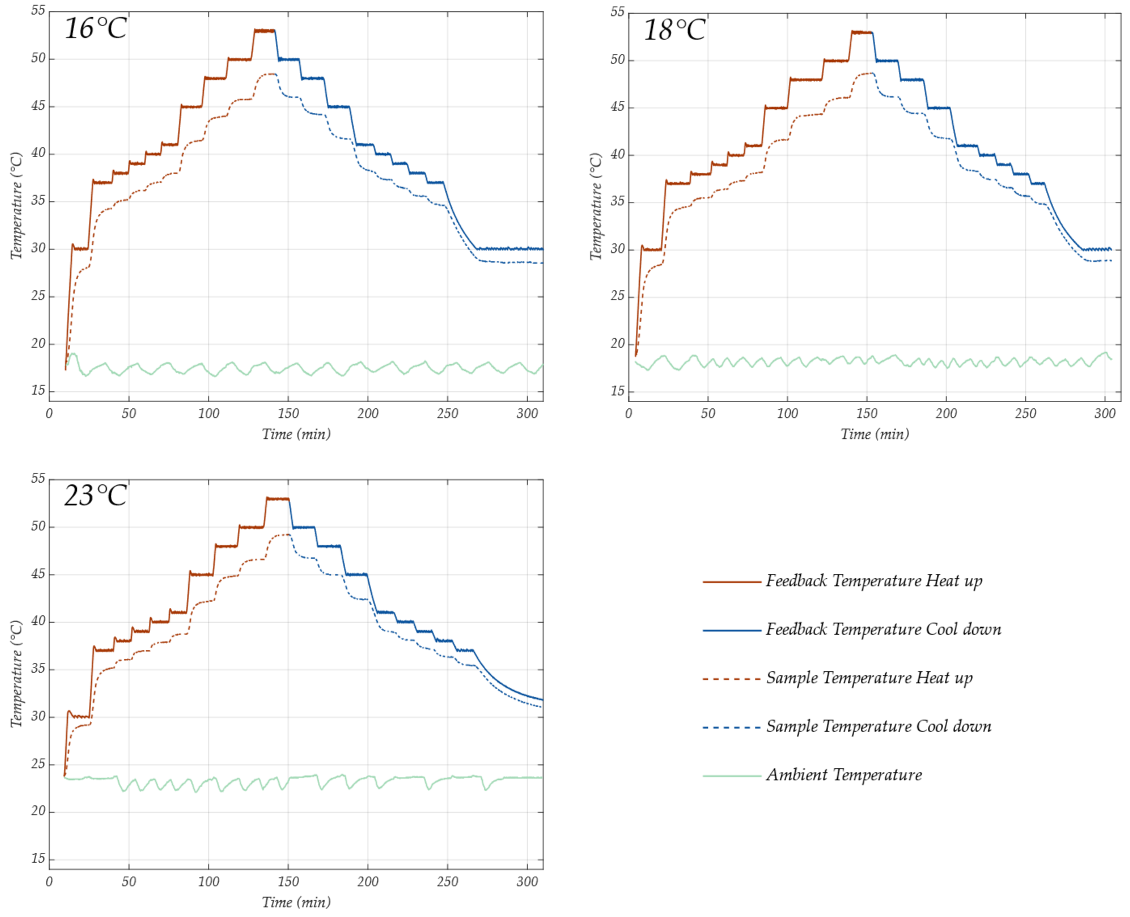

Figure S1: Recorded characterization data for feedback temperature control, sample position temperature and ambient (laboratory) temperature of 16, 18 and 23°C.

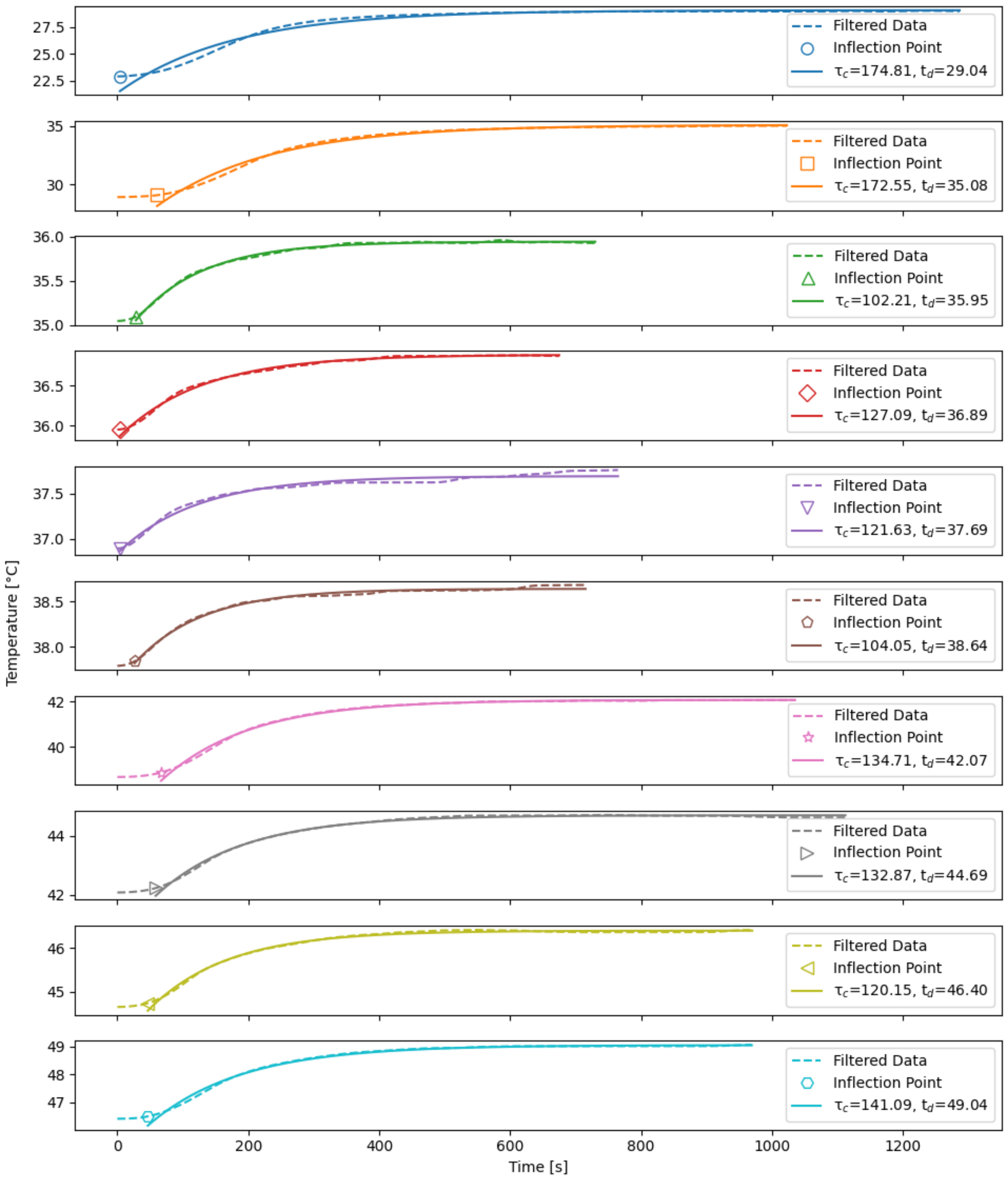

Figure S2: Exponential fit and time constant estimation of the standard deviation cured heating steps in the temperature characterization data (21°C) from Figure 3 in the main article.

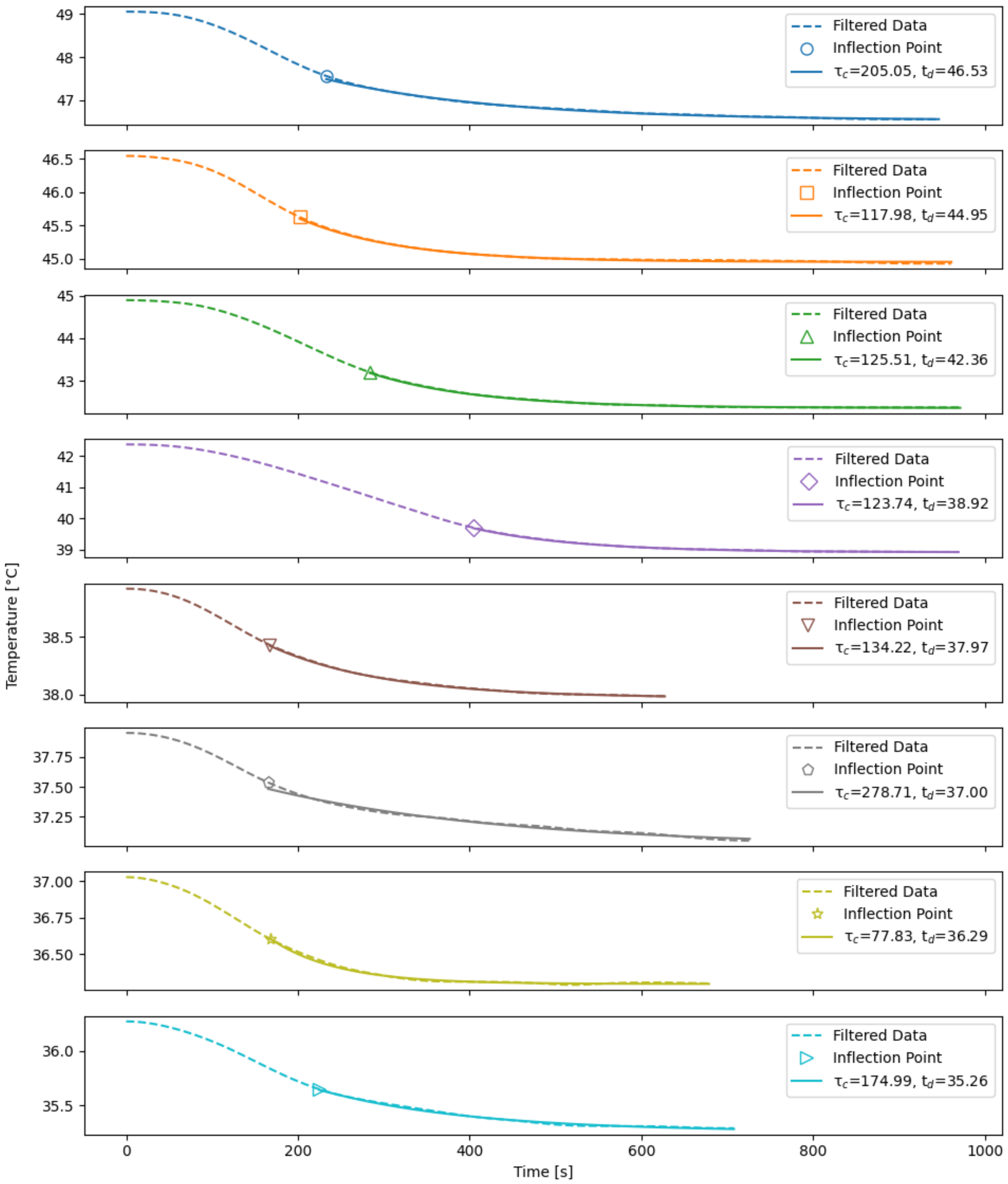

Figure S3: Exponential fit and time constant estimation of the standard deviation cured cooling steps in the temperature characterization data (21°C) from Figure 3 in the main article.

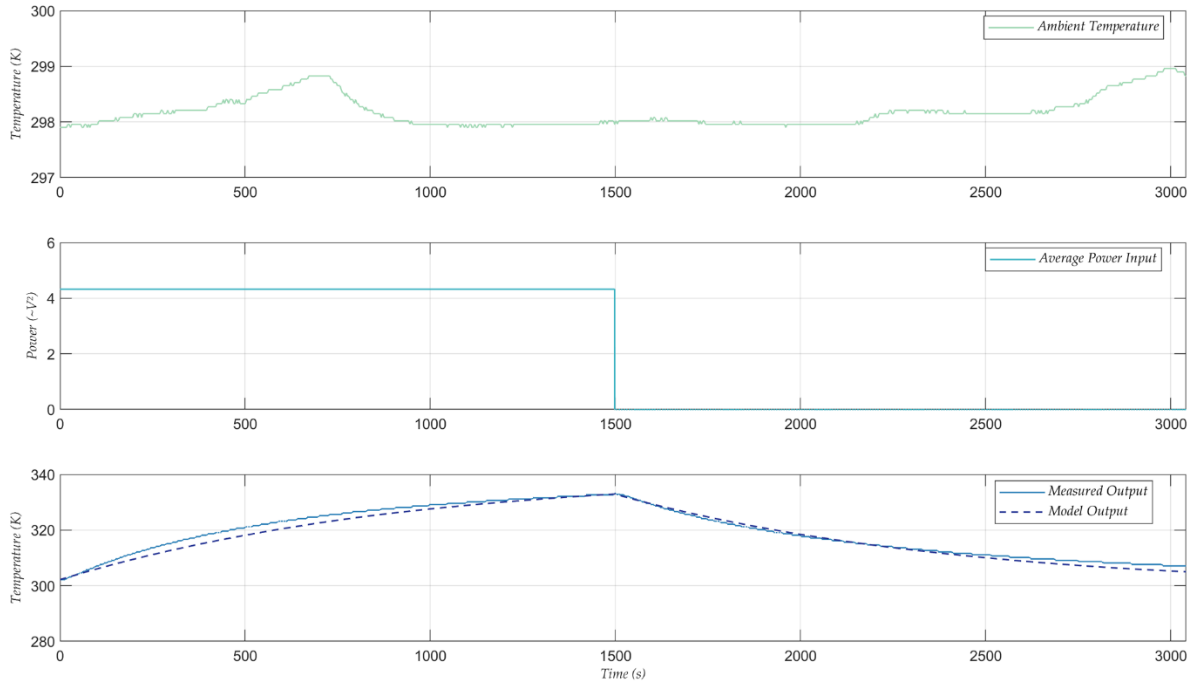

Figure S4: System in- and outputs used as model structure identification data for the lumped parameter model that predicts temperatures at the feedback sensor position. The corresponding model parameters can be found in the main article.

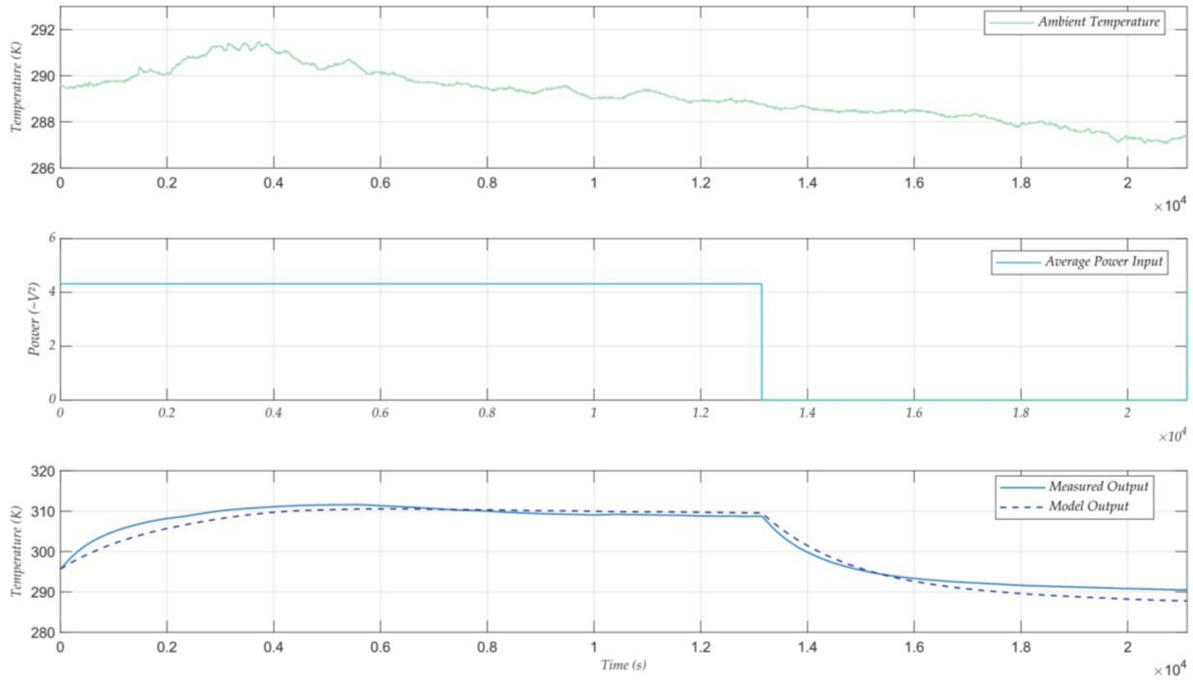

Figure S5: System in- and outputs used as model structure validation data for the lumped parameter model that predicts temperatures at the feedback sensor position. The corresponding model parameters can be found in the main article.

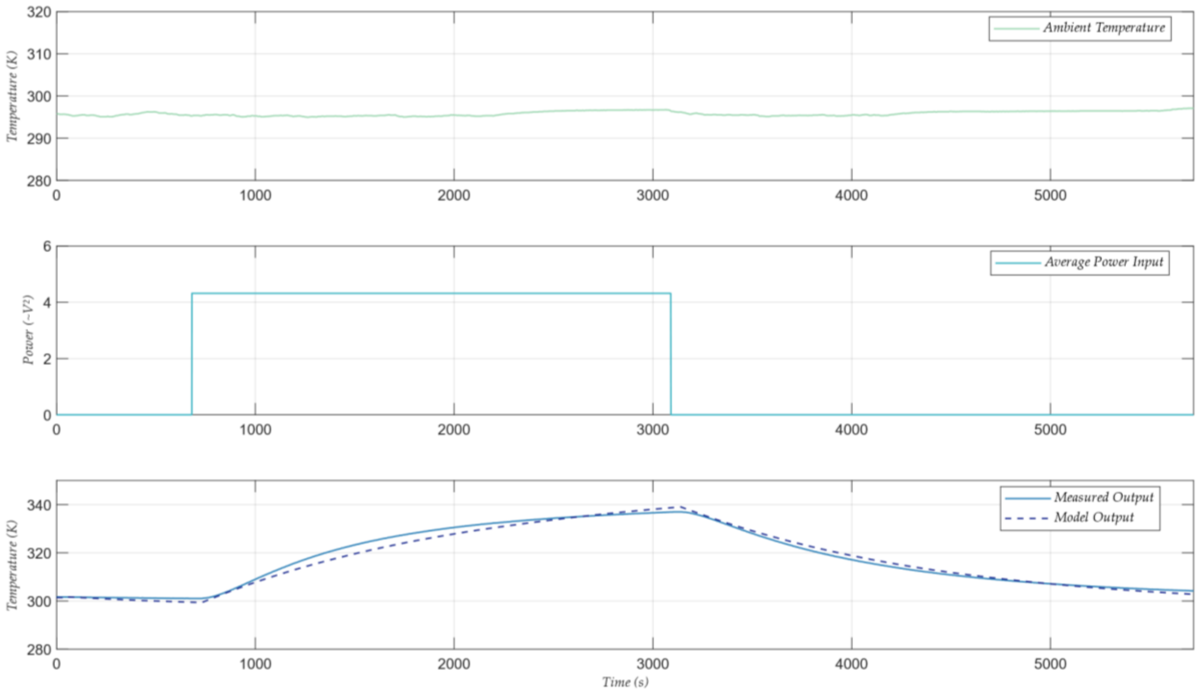

Figure S6: System in- and outputs used as model structure identification data for the lumped parameter model that predicts temperatures at the sample position. The correspondingly used model parameters can be found in the main article.

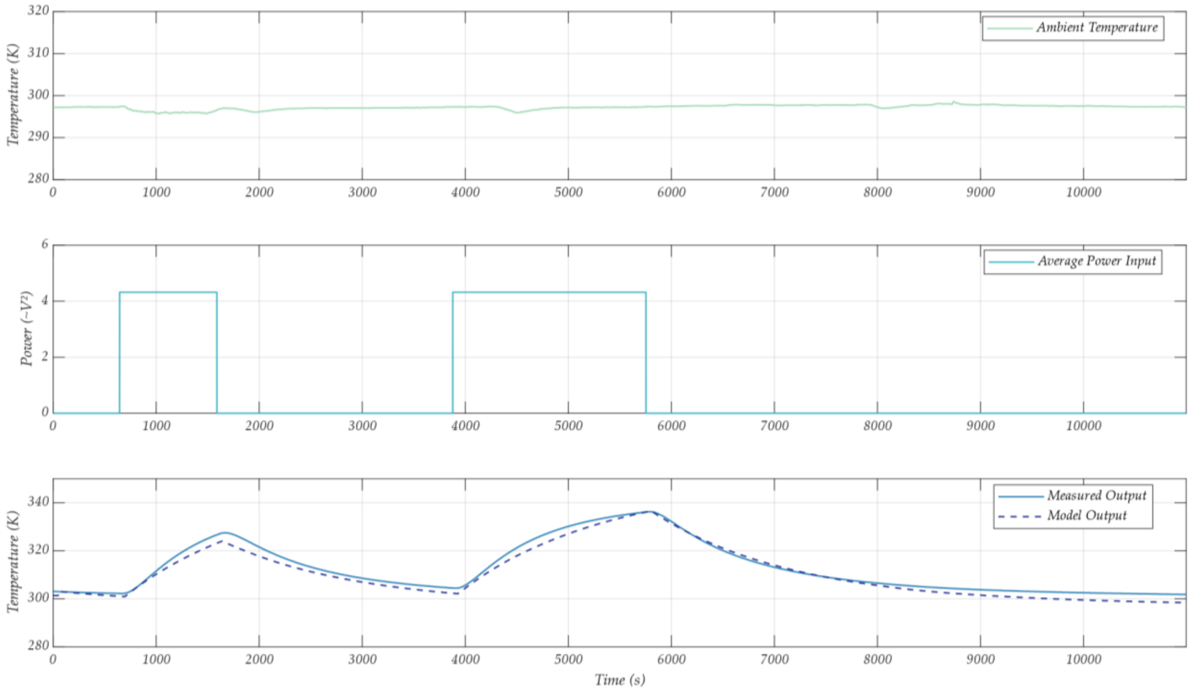

Figure S7: System in- and output used as model structure validation data for the lumped parameter model that predicts temperatures at the sample position. The corresponding model parameters can be found in the main article.

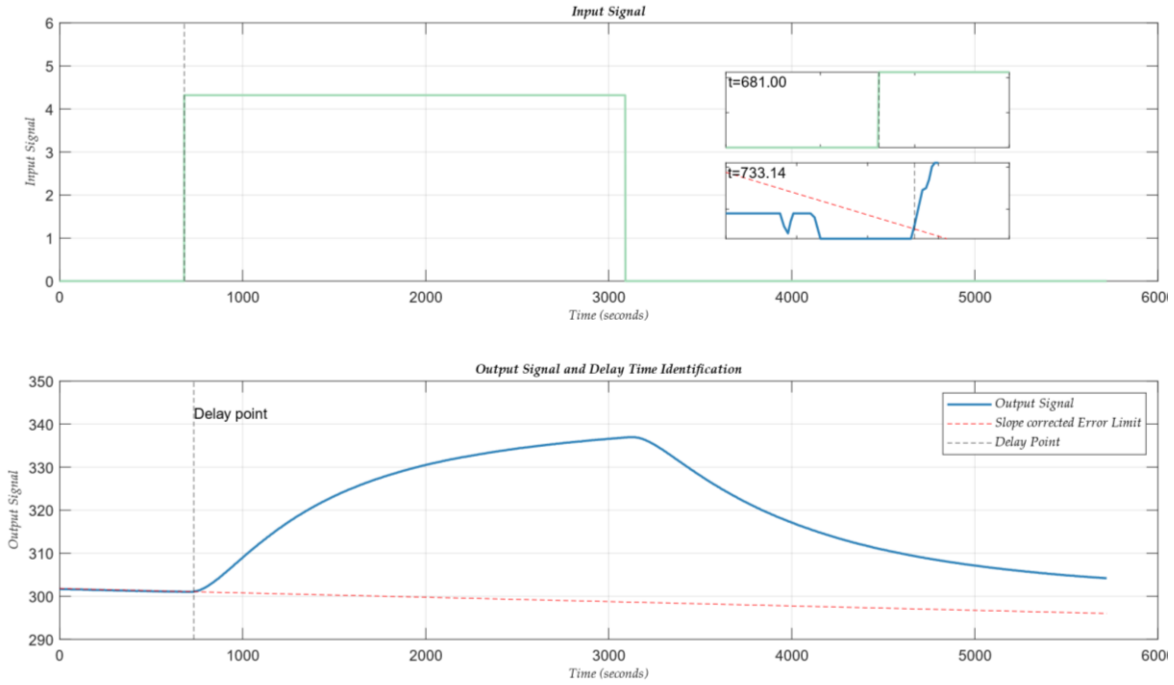

Figure S8: Delay time estimation for system identification dataset. The delay time is the difference of the rising edge of the input pulse and the first significant change in the output temperature.
